# Dialogic literary argumentation and close reading: effects on high school students’ literature-related argumentative writing and motivational beliefs

**DOI:** 10.3389/fpsyg.2023.1214773

**Published:** 2023-07-31

**Authors:** Kevin Fulton, Tzu-Jung Lin, George Newell

**Affiliations:** ^1^Department of Educational Studies, The Ohio State University, Columbus, OH, United States; ^2^Department of Teaching and Learning, The Ohio State University, Columbus, OH, United States

**Keywords:** close reading, dialogic literary argumentation, writer beliefs, writing self-efficacy, literature-related argumentative writing

## Abstract

Given evidence that adolescent students’ motivation to read and write about literature declines with age, we proffer an approach called dialogic literary argumentation (DLA) that asks students to explore literature through argumentation in pursuit of understanding the meanings and possibilities of being human. This quasi-experimental study compared the effectiveness of DLA with close reading (CR), a common approach to teaching literature in high school English language arts classrooms, in improving students’ motivational beliefs about writing and literature-related argumentative writing. The study also examined how the links between motivational beliefs and argumentative writing performance varied by instructional contexts. Participants were 278 high school students in 14 classrooms across 8 public high schools. Classrooms of students received either DLA or CR throughout the academic year. While both the DLA and CR groups improved in literature-related argumentative writing, the DLA group demonstrated more growth than the CR group. Neither group exhibited changes in motivational beliefs. However, at the end of the year, both DLA and CR students’ transactional writer beliefs were predictive of writing self-efficacy. Transmissional writer beliefs negatively correlated with argumentative writing in the CR group and had a null relationship in the DLA group. Overall, motivational beliefs and argumentative writing were more positively correlated in the DLA group than the CR group after the intervention. We posit that the argumentative elements unique to DLA may act to protect students from the negative impacts of transmissional beliefs. Our findings provide theoretical explanations and pedagogical recommendations on how DLA and CR can be jointly employed to heighten students’ motivation and strengthen their argumentative writing competence.

## Introduction

Although conceptualized differently according to discipline or theoretical framing, common features of argumentative writing often include a well-reasoned claim, with relevant evidence, warrants, and occasionally counterarguments, or rebuttals (Toulmin, 1958). Teachers, scholars and other professional educators have viewed argumentation and argumentative writing as an important literacy skill and academic practice necessary for students to become part of a democratic citizenry. Specifically, the teaching and learning of argumentative writing is important to study in part because it is an academic and practical life skill students will repeatedly make use of in and out of schooling (Graham and Perin, 2007; Shanahan and Shanahan, 2008).

Reviews of research and the experiences of teachers and scholars alike have revealed that the dominant approach to teaching and learning argumentative writing in US schools consists of slotting information into preexisting forms of the Toulmin elements (Hillocks, 2005; Newell et al., 2011; Campbell and Latimer, 2012). Writing scholars have argued that this dominant model may limit writers (DeStigter, 2015) or promote binary thinking (Newell et al., 2015). Perhaps because the approach is driven by test-preparation rather than as a way to communicate ideas to a reader, many high school students do not feel motivated or confident to write (Pajares et al., 2007). Research further suggests that adolescent students’ writing motivation and their relationships with writing performance decline over the school years (Boscolo and Gelati, 2019; Camacho et al., 2021). This decline is concerning because writing motivation beliefs play an influential role in the quality and amount of writing produced (Troia et al., 2013; Graham et al., 2017). Unfortunately, little instructional time is devoted to teaching writing in elementary (Gilbert and Graham, 2010) or high school (Applebee and Langer, 2013), making it more difficult for teachers and researchers to address these concerns.

Our scholarly interest is focused on secondary English language arts (ELA) classrooms, a content area in US secondary schools focused on reading and writing with a large focus on literary texts (Applebee, 1993). The teaching of literature is defined not only by the choice of texts to teach but equally important are questions regarding what teachers do to support and guide students’ readings of those texts and how they assess what students have learned. A reading of, say, a Langston Hughes poem that raises students’ experiences with unfairness and racism is a very different reading from the same poem that focuses on reading comprehension or techniques of literary analysis.

The most recent 2019 National Assessment of Educational Progress (NAEP) included results for grades 4, 8 and 12 showed that the average literary text comprehension score was lower in 2019 than in 2015 overall. Perhaps just as concerning is the seeming decline in motivation to read literature. In 2019, 26% of all twelfth graders in the nation reported that they never read stories or novels, and 51% of twelfth graders reported that they never read poems outside of school. Larger percentages of lower-performing students (below the 25th percentile) than higher-performing students (at or above the 75th percentile) reported never reading these types of literary texts.

Dialogic literary argumentation (DLA) and close reading (CR) stand in as potential answers to this educational quandary. Built upon our research over the past 15 years (Newell et al., 2015, 2018; Bloome et al., 2020), DLA was developed as a framework for the teaching, learning, reading, and writing about literature. It asks students to read and write about literary texts with an open mind and to engage in dialogue with others using the literature they have read to explore what it means to be human. DLA begins with the assumption that the role of dialogic argumentation as a social practice is to shape students’ and teachers’ understanding of literary texts, the human condition, and the complex social world. Research suggests that argumentation can increase engagement (Chinn et al., 2001), motivation (Chinn, 2006), and written arguments (Deane and Song, 2015), and theorizes that dialogic pedagogical approaches can lead to increased student motivation (Matusov, 2009).

The most recent study of literature instruction in US secondary schools, revealed that close reading (CR) is a common practice in high school classrooms (Applebee, 1993). Although defined differently across studies and pedagogical approaches (Catterson and Pearson, 2017), CR has the potential to improve student writing (Dollins, 2016). For our purposes, we follow Brown and Kappes (2012, p. 2) and the Aspen Institute to define CR as “an investigation of a short piece of text, with multiple readings done over multiple instructional lessons.”

However, our interest in the effectiveness of CR and DLA is not concerned with assessing which “works better” but to consider how they may be employed jointly to motivate students and to deepen their ways of responding to and understanding issues, ideas and themes in literary texts. To our best knowledge, research that integrates DLA and CR has not been done to this day. Note too that we agree with Catterson and Pearson (2017) that despite all the talk and concern about close reading, “[research] findings lack the sort of specificity needed to make precise pedagogical recommendations” (p. 470). We see our work as a single step in the direction of their suggestion and for good reason.

Our study examines the relationships between motivational beliefs about writing and literature-related argumentative writing in high school English language arts classrooms. We explore the effects of DLA on high school students’ motivation to write and performance on argumentative writing in comparison to an active control-comparison approach called CR. Our central hypothesis is that DLA would demonstrate added values to the CR approach based on its impacts on high school students’ motivational beliefs and literature-related argumentative writing. Specifically, compared to students who were taught using a CR approach, students receiving the DLA instruction would experience more positive changes in writing motivation and in their performances of literature-related argumentative writing, and demonstrate stronger linkages between writing motivation and the quality of argumentative writing. This study provides the first set of quantitative findings on the relative effectiveness of DLA instruction and close reading for students in high school ELA classrooms.

## Research background

### Dialogic literary argumentation

Dialogic Literary Argumentation (DLA) is a framework grounded in social practices and processes for teaching and learning to write literary arguments. This includes discovering and exploring complex ideas that values and that respects multiple perspectives, shifts social relationships from competitive to collaborative roles, and requires redefining knowledge as situated, multiple, and continuously evolving. Simply put, rather than positioning the teacher as transmitter and students as recipients of full formed literary interpretations, dialogue in DLA classrooms can take place with an open mind and take up argumentation as a social practice and process of learning with others and with literature (Seymour et al., 2020).

One way DLA seeks to foster a better understanding of the human condition and the text itself is by utilizing argumentation as an inquiry and learning strategy (Newell et al., 2015). Importantly, rather than emphasizing argumentative writing as a way to demonstrate a final analytic performance of synthesizing texts and ideas, a tradition of writing-to-learn research has offered students an opportunity to think analytically and to learn about the content of writing through composing (Langer and Applebee, 1987; Scardamalia and Bereiter, 1987). The DLA approach reframes argumentation as less than a way to present full-formed ideas and more as arguing-to-learn in which students and teachers use argumentation as a social means for exploring and examining their social worlds (Newell et al., 2015).

As an approach to literature instruction, DLA has teachers take on a dialogic stance. Rather than taking on ideas blindly, students are encouraged to understand how their ideas exist in relation to others’ ideas for the purposes of learning and understanding the world more fully. In addition, DLA requires students to have open conversations with differing, enriching perspectives about the text and its connections to their lives. The primary goal is to bring to their interactions textual evidence that includes the targeted literary texts as well as ideas from prior discussions, previously read literature and narratives from and about students’ own experiences, as well as from their communities among others (Bloome et al., 2020).

While consensus may develop, it is not the goal of argumentation; the goal is to lead to “learning, growing, appreciating complexity, valuing diverse perspectives and experiences, and increasing acumen in arguing-to-learn are the goals” (Bloome et al., 2020, p. 38). This skill has always been important in a democratic society (Dewey, 1916), but it is particularly important today as society and politics become increasingly divided (Iyengar et al., 2012) and a majority of Americans have strong negative feelings toward those with different politics (Pew Research Center, 2022).

To develop a more informative dialectic, students engage in arguing to learn *via* a process of alternatively arguing (Newell et al., 2015). This is distinct from counterarguing because students are not aiming to win the argument; instead, alternative arguing is used to explore the text by adding layers of meaning and insight as students bring up new ideas in such a way that respects the text and recognizes the context of the individual (Bloome et al., 2020). For students to take up these meanings, they must engage with their peers’ alternative theses of the text and its relation to their own lives. As such, argumentation as learning requires students to consider more complex, multi-perspectival definitions of knowledge often focusing on race, class, gender, and sexuality due to their prevalence in classical and contemporary literature as well as their relevance to students’ everyday lives. This is designed to help them grow in their understanding that the human condition, including their own, is continuously changing [see (VanDerHeide et al., 2023) for a fuller explication of a DLA framework for teaching argumentative writing].

### Close reading

Close reading (CR) has been given various definitions and has been associated with various interpretations regarding its value in taking readers deeply into the processes of responding to the text itself as the central influence on student learning and comprehending literature (Brown, 2013; Fisher and Frey, 2015). Additionally, teachers have a wide range of interpretations and applications of close reading in their classrooms (Brown, 2013; Fisher and Frey, 2015). Broadly speaking, CR involves multiple re-readings of a shorter text selection with each reading focusing on a different technical aspect to better help students understand what the text means (Shanahan, 2012). A narrower definition of CR focuses solely on driving students to focus on “understandings that can be derived from analysis of the relationships and patterns found, as some have described it, within the four corners of the page” (Beers and Probst, 2013, p. 34).

In the current study, we follow Brown and Kappes (2012) definition that defines CR as an investigation of a short piece of text using multiple instructional methods, such as text-based questions and discussion, attention to form, tone, imagery and/or rhetorical devices, and word choice and syntax. The goal of CR is to bring the text and the readers *close* together through paying “close attention to the relevant experience, thought, and memory of the reader; close attention to the responses and interpretations of other readers; and close attention to the interactions among those elements” (Beers and Probst, 2013, pp. 36–37). Beers and Probst (2013) list key features of close reading as accomplished through multiple re-readings of a short passage with an intense focus on the passage itself. Readers draw out subjective feelings and thoughts that eventually return to the text to explore the words, events, ideas, and connections of the elements in the passage that, through exploratory discussions, extend to other parts of the text.

Fisher and Frey (2014) recommend that multiple re-readings can be spaced out over several lessons as students analyze different aspects of the text with text-dependent questions. The first reading can be done for “big picture” ideas, the second should focus on specific lesson goals such as author’s purpose or text structure. Subsequent re-readings should focus on shared read alouds whether the teacher models their thinking or on having students respond to text-dependent questions by locating evidence in the text. Text-dependent questions can be closed- or open-ended and are defined as questions where students meaningfully engage with the text to come up with an answer. These questions are scaffolded and can range from general comprehension questions to complex inference questions, and can even include opinion, arguments, and intertextual connection questions (Fisher and Frey, 2014).

The purpose behind this pedagogical choice is for students to draw meaning out of the text through the transaction between the reader and the text (Beers and Probst, 2013). To accomplish this, in close reading students should notice, question, and weigh things against their lives and the world. Beers and Probst developed signposts as a scaffolding strategy to allow students to independently conduct close reading of the text and to connect close reading to their own life experience, other texts, and world events. While students are able to do CR individually, over the course of a lesson they are also involved in dialogic conversations where they reflect, ask questions, and propose answers and explanations together. This can be fostered through carefully crafted text-dependent questions, answered through dialogic discussion with teachers and peers (Beers and Probst, 2015, p. 28–29). Discussion is seen as a key component of CR because it allows students to “engage in the interplay of ideas, some contradictory, that support reasoning” (Fisher and Frey, 2014).

### The added values of DLA to CR

Both DLA and CR involve the use of student-oriented, teacher-guided discussions to help students analyze the text. Discussions are centered around a key concept, question, or problem posed by the text. These discussions in DLA and CR tend to be more transactional than traditional lecture-based approaches to teaching and learning because they involve students’ active participation in the dialogic process of understanding the world around them (Applebee et al., 2003). Teachers provide students with numerous opportunities to talk, maintaining an egalitarian social dynamic in the classroom. However, one instrumental distinction between DLA and CR is that CR does not intentionally incorporate argumentation into classroom discussions about the text. DLA’s multi-perspectival approach encourages students to engage in arguing-to-learn where students support their arguments with claims, evidence, and warrants under the assumption that their argument will continuously change through engaging in dialogue with others (Bloome et al., 2020). Another key distinction is how the text is considered in each framework. Unlike CR, DLA does not view literary texts as worthy of study in and of themselves. Specifically, DLA views texts as “argumentative props” (Seymour et al., 2020, p. 29). Rather than a rejection of the text itself, however, DLA emphasizes the flexibility of how teachers and students might read and use literary texts to engage in their social worlds. Here the value of using literature as an argumentative prop opens the possibilities for students of differing experiences and perspectives. In this way, argumentation and argumentative writing become ways of taking social action to deepen analysis of the text and to understand perspectives of others.

To engage students in literary argumentation, questions posed within a DLA classroom are not only open-ended but involve some level of conflict based on controversial or socially contested topics to increase students’ engagement and motivation to consider multiple perspectives. Effectively engaging in these conversations and writings requires risk taking and trust by and between teachers and students (Seymour et al., 2020). Teachers take a risk by relinquishing a level of control and giving students the space to compose interpretations with depth and nuance. Students take a risk when they engage in argumentation because these the questions involve ambiguity and uncertainty. And even though the teacher is giving students freedom to have these conversations and compositions, students still need to trust that the teacher will provide ongoing support, beyond the initial prompt.

One way DLA teachers create an environment where these conversations and compositions are more likely to be successful is in fostering a class culture surrounding the arguing-to-learn approach that is supportive, understanding, and collaborative. Fostering an arguing-to-learn class culture where students are supportive, understanding, and collaborative plays a central role in increasing the success of these conversations and compositions. Without this, discussing controversial topics can become adversarial and rude, often creating a vicious cycle (Chiu and Khoo, 2003). DLA teachers address these concerns by embracing the tensions in order to more deeply explore them with others.

Another tangible difference between CR and DLA is that DLA has an explicit expectation that students will make connections to their daily lives as they explore what the text can reveal about the human condition, often using personally relevant topics such as race, class, gender, and sexuality as productive analytical lenses (Bloome et al., 2020). DLA’s focus on the human condition and multiple perspectives helps students go beyond themselves and their own experiences by incorporating others’ perspectives with their own leading to a more informative dialectic. This contrasts with CR’s approach where students may use the text to connect to their own lives and world events (Beers and Probst, 2013) albeit, in a much more individualistic sense.

### Motivational beliefs about writing

Although DLA has been developed through collaboration with over 60 teachers through 15 years of collaboration (Newell et al., 2015), research has yet to assess how, when, and why DLA may influence student motivation. Similarly, even though CR has been incorporated in the Common Core State Standards for over a decade (Hodge et al., 2020), CR has not been systematically studied, and individual empirical studies are difficult to find (Hinchman and Moore, 2013). Any effects CR may have on student motivation are largely understudied, despite some anecdotal evidence or theoretical arguments (Brown and Kappes, 2012). While neither DLA nor CR have been studied in regard to student motivation, both theoretically could influence student motivation.

According to Writer(s)-within-Community Model Graham (2018), motivational beliefs about writing include beliefs about the utility and value of writing, motives for writing, attitudes and interests towards writing, beliefs about writing successes or failures, self-efficacy for writing, reasons for writing, writer’s identities, and beliefs about the communities in which writing occurs. Writing is simultaneously shaped by these motivational beliefs along with other cognitive capacities of individuals, as well as resources and capacities of the community. In the current study, we specifically focused on two types of motivational beliefs: self-efficacy for writing and argumentative writing, and writer’s transactional and transmissional beliefs as a form of writers’ identity. We chose to study writers’ self-efficacy for writing and argumentative writing because it directly aligns with our study goal to enhance students’ writing competence. It is also the most researched motivational belief in writing research, as reviewed below. We chose to study transmissional and transactional writer beliefs because the Dialogic Literary Argumentation instruction is centered on the social practice perspective that strongly aligns with the transactional writer beliefs. If students demonstrated a positive change in the writer’s belief, it would strongly support the effectiveness of the DLA instruction.

Self-efficacy for writing is defined as a learner’s perceived ability to write (Martinez et al., 2011; Bruning et al., 2013). It has been a strong predictor of self-regulation for writing (Zimmerman and Risemberg, 1997; Paul et al., 2021) and writing performance (Pajares, 2003; Bruning and Kauffman, 2016; Graham et al., 2017; Graham, 2018). According to the social cognitive theory (Bandura, 1977, 1997), students with high self-efficacy for writing are more likely to engage in cognitive and behavioral regulation processes of writing such as goal setting, monitoring and evaluating goal process, and creating effective environments that result in improved writing (Schunk and DiBenedetto, 2016). As the student continues to write, they receive self-feedback and external feedback on their progress; when they believe they are making positive progress, their self-efficacy increases and leads to better writing performance (Graham, 2018).

Despite the fruitful amount of evidence supporting the relationship between self-efficacy for writing and writing performance, few of the studies have examined whether such a relationship is genre specific. Writing genres differ by their communicative goals, sociocultural practices, roles, and skills (Ravid and Tolchinsky, 2002). Narrative writing, for example, requires that the writer describes events with a focus on people and their actions (often in response to a crisis) that unfold over a period of time in specified locations. Argumentative writing focuses on making a reasoned, justified argument about an unresolved and oftentimes controversial issue. Among different genres of writing, argumentative writing has been considered a complex genre to teach and learn (Jagaiah et al., 2020). Differing rhetorical demands and purposes between writing genres raise a question about whether the association between self-efficacy and writing performance applies broadly across writing genres (Hidi et al., 2002). This motivated us to measure and triangulate between self-efficacy for writing (genre-general) and self-efficacy for argumentative writing (genre-specific) to identify any genre-specific patterns in our findings.

In addition to self-efficacy for writing, students’ beliefs about writing, hereafter called writer beliefs, can influence their writing process and the writing outcome (Graham et al., 1993). Writer beliefs shape writer’s actions (Pajares and Johnson, 1996). Two particular sets of writer beliefs that are the foci of our analysis are transmissional and transactional writer beliefs. Transmissional writer beliefs assume that meaning exists independently of the writer and writing is transmitted from sources of reading to the writer (Baaijen et al., 2014). Conversely, transactional beliefs assume that meaning is actively constructed by the writer (Schraw and Bruning, 1999). The two types of writer beliefs have been shown to orthogonally relate to each other (Mateos et al., 2011), suggesting that each of the beliefs can independently shape students’ understanding of their roles as the writer in an instructional context, orienting them to approach the tasks of writing in particular ways (White and Bruning, 2005).

As transmissional writer beliefs encourage students to view meaning as external, such beliefs tacitly encourage writers to passively engage with writing with lower levels of affective and cognitive engagement (White and Bruning, 2005). High transmissional writer beliefs have been associated with prioritizing “objective” facts without the writer expressing their own point of view, putting the writer in a bind as it becomes more difficult to express their own thoughts (Baaijen et al., 2014). Affectively, transmissional writing beliefs have been associated with greater writing apprehension, grammar apprehension, and lower self-efficacy for writing (Sanders-Reio et al., 2014). In school, writers with high transmissional beliefs, who believe writing is about citing authorities, produced lower quality text than writers with low transmissional beliefs (Baaijen et al., 2014).

In contrast, high transactional beliefs have been associated with better writing quality than low transactional beliefs (White and Bruning, 2005). Students who have high transactional writer beliefs may be more intrinsically motivated to express their own ideas in writing arising from content learning, background knowledge, and through the process of revising (Baaijen et al., 2014). High transactional writer beliefs have been associated with greater levels of enjoyment, reduced writing apprehension in writing (Sanders-Reio et al., 2014) and higher self-efficacy for writing (White and Bruning, 2005).

### The current study

In this study, high school students received either a DLA or a CR approach to teaching literature-related argumentative writing during their English language arts sessions throughout an academic year. The purpose of this study was to compare differences in motivational beliefs (writer beliefs and self-efficacy for writing) and literature-related argumentative writing performance, as well as the links between motivation and writing, between students in the DLA classrooms with students in the CR classrooms. We addressed two research questions. First, how do students in the DLA and CR classrooms differ in their writer beliefs, self-efficacy for writing and literature-related argumentative writing, and argumentative writing at the post-test, controlling for baseline differences and student characteristics (gender, grade level, academic track)? Second, at the end of the academic year, how do the relationships between motivational beliefs and literature-related argumentative writing differ between the DLA and CR groups, controlling for gender, grade level, and academic track?

Our working hypotheses are that students taught using the DLA approach throughout the academic year would demonstrate higher transactional writer beliefs, lower transmissional writer beliefs, greater self-efficacy for writing/argumentative writing, and higher literature-related argumentative writing performance than students experiencing CR, showing the added values of DLA to CR. For the second research question, we hypothesize that literature-related argumentative writing performance would be positively correlated with self-efficacy for writing/argumentative writing and transactional writer beliefs, and negatively correlated with transmissional writer beliefs. Similarly, self-efficacy for writing/argumentative writing would be positively correlated with transactional writer beliefs and negatively correlated with transmissional writer beliefs. With the caution that our findings would be correlational and not causal, we explored the mediating role of self-efficacy for writing/argumentative writing between writer beliefs and literature-related argumentative writing performance. Since transactional writer beliefs are more aligned with DLA’s design principles than transmissional writer beliefs, we predicted that transactional writer beliefs would be more correlated with self-efficacy for writing/argumentative writing and literature-related argumentative writing performance in the DLA group than in the CR group. The associations between transmissional writer beliefs, self-efficacy for writing/argumentative writing, and literature-related argumentative writing performance would be null or negative for both the DLA group and the CR group.

## Methods

### Participants

This quasi-experimental study was conducted during the piloting phase (Year 3) of a four-year project (2016–2017 school year) focusing on developing and implementing a principled approach to teaching and learning literature to effectively support students’ literature-related argumentative writing. Participants included 278 high school students (47.1% female) in 14 classrooms (2 in 9th grade, 5 in 10th grade, 7 in 11th or 11th/12th grade) from eight schools across six school districts in the Midwestern United States. In terms of students’ race and ethnicity, 63.7% (*n* = 177) of the students were White, 12.6% (*n* = 35) were Black, 2.5% (*n* = 7) were Asian, 2.2% (*n* = 6) were Hispanic, 1.4% (*n* = 4) were Native Hawaiian or Pacific Islanders, 7.6% (*n* = 21) were multi-racial, and 10.1% (*n* = 28) were missing. About 5.8% of students reported speaking languages other than English at home. Five of the classrooms were Advanced Placement (AP) classrooms focusing on either literature or written composition, while the other classrooms were “college preparation” (CP) academic level classrooms. The 14 teachers were selected based on recommendations by building principals, their respective English department chairs, and university-based teacher education professionals.

### Study design and procedure

Prior to the quasi-experimental study, during school years 2014–2015 and 2015–2016 we carried out multiple design-based research and development projects based on the principles and practices of social practice theory (Gee, 1990; Street, 1993; Newell et al., 2015) with 13 collaborating teachers across Year 1 and Year 2 to iteratively design, refine, and adapt a feasible and effective intervention on literature related argumentative writing that we refer to as a “Dialogic Literary Argumentation” approach. In summer 2014, summer 2015, and summer 2016 during Summer Workshops, in collaboration with ELA high school teachers we developed exemplary curricular units, including formative assessments for high school ELA classrooms. We also met with these teachers monthly to not only articulate their developing approaches to literature-related argumentative writing but also to garner support for some of the challenges they face in introducing argumentation into the study of literature.

During school year 2016–2017 we collected more formal, pilot study data to determine whether the Dialogic Literary Argumentation intervention was operating as intended to change students’ learning opportunities and outcomes, with particular concern for high quality performance of literature-related argumentative writing. Eight teachers were recruited to participate in the DLA group, and six teachers in the CR group. During summer 2016 we held separate teacher workshops for the DLA and CR groups. During the workshop with the DLA teachers, we reviewed the principles of the curricular intervention and then asked the teachers to develop ideas for instructional plans shaped by those principles. During the workshop with the CR teachers, we reviewed the principles of close reading based on the conceptualization proffered by Beers and Probst (2013) and engaged them in practicing the uses of “signposts” or moves fiction authors make in literary texts taught in middle school and high school language arts classrooms.

During the Summer Workshops, we also met with teachers from both groups individually to support curriculum plans for each teacher’s target classroom. At the end of each workshop, the teachers and the research team met as each teacher presented his or her curriculum plan. We then met with all teachers in an additional meeting just before school districts opened for the 2016–2017 school year to discuss research procedures and design issues for implementation and observation of the enactment of the curriculum in each classroom.

By May 2017, we had met with all teachers and provided ongoing support in separate groups about 5 times with each meeting lasting about 90-min. At these teacher meetings we had each teacher report-out “how things are going.” With the DLA group teachers, we also discussed two on-going ways to frame literary argumentation: learning to argue and arguing to learn with particular attention to how these ways of framing argumentation might evolve across the school year. We also studied how the DLA teachers enacted the principles and practices of our curricular intervention that we refer to as an “Dialogic Literary Argumentation” approach to ensure the inclusion of teachers’ ideas in its formative development.

During CR teacher meetings, we discussed the transition from teaching students the signposts (Beers and Probst, 2013) of close reading (e.g., contrasts and contradictions, etc.,) to teaching students how to use the signposts as text-analytic tools for close reading of literature, centering on noticing and interpreting author’s intentions, individuals’ sense-making processes, and the structural aspects of argument/argumentation. At these meetings, each teacher reported-out “how things are going.” In this way, the teachers not only articulated their developing approaches to close reading of literature but also garnered support for some of the challenges they faced in introducing close reading into the study of literature. We also discussed two on-going ways to frame close reading: learning the signpost of close reading and learning to use the signposts for close reading.

As an alternative to a fidelity measure of the 14 teachers’ instruction, we conducted extensive classroom observations of the teachers across the 2016–2017 school year grounded in microethnographic discourse methods (Weyand et al., 2018). This approach allowed us to consider the teachers’ instructional principles as grounded in either DLA or CR. Each teacher was observed by a field researcher. To ensure that the teachers in each condition relied on the instructional principles of either DLA or CR, the field researchers collaborated with their case study teachers in planning the instructional units. They observed each classroom several times (n = 63 observations across 8 DLA teachers; n = 44 observations across 6 CR teachers) to learn how those activities were carried out. These activities were planned as a school-university collaboration in which the various participants contributed their particular expertise to the ongoing work.

### Measures

Each of the measures below were administered at the pre- and post-tests. Students were instructed to indicate how true each response was to them on a 5-point Likert scale (1 = not at all true, 5 = always true). Item reliability was reported using Cronbach’s Alpha. To determine if the measures of writer beliefs and writing self-efficacy function comparably for the DLA and CR groups (in preparation for the multigroup analysis), we examined measurement invariance of writer beliefs and writing self-efficacy. Three types of measurement invariance were examined: configural invariance, metric invariance, and scalar invariance (van de Schoot et al., 2012). The measurement invariance test was conducted using the MODEL = CONFIGURAL METRIC SCALAR function under a multigroup confirmative factor analysis framework in Mplus. Based on the results of item reliability and measurement invariance tests, we identified reliable items and used them to compute composite scores of writer beliefs and writing self-efficacy for multiple regression and structural equation modeling analyses.

#### Writer beliefs

The writer beliefs scale was adopted from White and Bruning (2005). It consists of two subscales: transactional beliefs (9 items: “I enjoy interpreting what I read in a personal way.”; Pre-test α = 0.730, Post-test α = 0.802) and transmissional beliefs (10 items, “The main purpose of reading is to understand what the author says.”; Pre-test α = 0.718, post-test α = 0.800). We removed redundant items based on modification indices and retained five items under each subscale. As shown in Table 1, the two-factor model with the final items resulted in fair to good model fits for the configural models at both pre- and post-tests (i.e., CFI > 0.90, RMSEA <0.08) (Yuan et al., 2016). The metric invariance models at the pre-test fit the data slightly better than the configural model (based on the increased CFI and the decreased RMSEA), and model fit indices were comparable between the metric and scalar invariance models. At the post-test, CFI and RMSEA did not change much ( ΔCFI≤0.01,ΔRMSEA≤0.015) between the configural and metric invariance models and between the metric and scalar invariance models at the post-test. We therefore concluded the writer beliefs measure met the measurement invariance criteria. For the later analyses, we calculated the composite scores of transactional beliefs and transmissional beliefs based on the final items.

**Table 1:** Fit statistics from a set of multigroup CFAs run on the items of writer beliefs and writing self-efficacy to evaluate measurement invariance.

Group of Models	# of param	$\chi^{2}$			CFI	RMSEA	Model comparison	ΔCFI	ΔRMSEA
Stage of MI evaluation		Est	*df*	*p*					
T1 writer beliefs									
Configural invariance	62	92.48	68	0.03	0.93	0.05			
Metric invariance	54	93.12	76	0.09	0.95	0.04	With configural	0.02	−0.01
Scalar invariance	46	102.80	84	0.08	0.95	0.04	With metric	<0.00	<0.00
T2 writer beliefs									
Configural invariance	62	89.55	68	0.04	0.96	0.05			
Metric invariance	54	103.88	76	0.02	0.95	0.06	With configural	−0.01	0.01
Scalar invariance	46	116.49	84	0.01	0.94	0.06	With metric	−0.01	<0.00
T1 writing self-efficacy									
Configural invariance	68	131.26	86	0.001	0.96	0.06			
Metric invariance	59	143.13	95	0.001	0.96	0.06	With configural	<0.00	<0.00
Scalar invariance	50	152.37	104	0.001	0.96	0.06	With metric	<0.00	<0.00
T2 writing self-efficacy									
Configural invariance	68	141.61	86	<0.001	0.94	0.08			
Metric invariance	59	162.04	95	<0.001	0.93	0.08	With configural	−0.01	<0.00
Scalar invariance	50	173.09	104	<0.001	0.92	0.08	With metric	−0.01	<0.00

T1 = Pre-test, T2 = Post-test.

#### Self-efficacy for writing/argumentative writing

The self-efficacy for writing scale was adopted from Kaplan et al. (2009) and Prat-Sala and Redford (2010). It consists of two subscales: self-efficacy for writing (6 items: “I can compose a strong conclusion for an essay.”; Pre-test α = 0.88, post-test α = 0.85) and self-efficacy for argumentative writing (5 items: “While writing an essay, I can think of evidence against contrasting ideas without using personal opinions.”; Pre-test α = 0.82, post-test α = 0.80). As shown in Table 1, the two-factor writing self-efficacy model had a fair to good model fits for the configural models at both pre- and post-tests. CFI and RMSEA did not differ significantly between the configural, metric, and scalar models, suggesting that the two-factor writing self-efficacy measure met the measurement invariance criteria. Composite scores of self-efficacy for writing and self-efficacy for argumentative writing were calculated.

#### Literature-related argumentative writing

Two literature-related argumentative writing tasks were administered in the fall (September–October) and spring (April–May) of the 2016–2017 academic year. Each writing task required students to read a short fictional narrative and respond to a writing prompt that asked students to justify their interpretation of the literary text with a “well-crafted” argument. The order of the literary texts was fixed. At the pre-test, students read and argued about *The Story of an Hour* (Chopin, 1894/1976). The title of this story refers to the time elapsed between the moments at which the protagonist, Mrs. Mallard, hears that her husband is dead, and when she discovers that he is alive after all. After a quiet celebration, this turn of events leads to her sudden death, perhaps due to surprise and shock. At the post-test, students read and argued about the story of *War* (Pirandello, 1994/1918). War is set in a train carriage in Italy during World War 1. While their nation is at war with the Central Powers, the passengers worry about the loss of their sons. As the passengers describe their frustrations and anxieties, a man counters with the importance of patriotic sacrifice, but in doing so realizes his own sense of loss. Both stories were piloted during the development and field-testing phase of the project in Year 1 and Year 2. The stories were randomly distributed to a total of 307 students at the beginning of the academic year. Based on the scoring rubric described below, the student essays were scored by two professional raters who achieved a satisfactory inter-rater reliability (α = 0.80) based on 20% of the total essays. An analysis predicting students’ beginning-of-the-year argumentative writing performance by story type suggested no significant story effect (*p* = 0.21).

#### Assessment of literature-related argumentative writing

A primary trait scoring rubric for evaluating the quality of students’ performance of a literary argument (Table 2) was developed based on Marshall (1987) and Newell (1994) studies of literary understanding and theories of argumentation (Toulmin, 1958). The rubric contained three levels of literary argumentation: evaluation, retelling, and interpretation. Evaluation refers to the writer’s judgement of the quality of the work, character’s behavior, idea (“War is always bad.”) or author’s vision of the world (“The author seems to think that all people are stupid.”) without explanation or justification and was considered as an off-task response to the literary text. Retelling refers to the writer summarizing the text with or without interpretive tags (elements suggestive of interpretation without integrating it into the essay to make the interpretation significant or central to the meaning of the essay).

**Table 2:** Scoring Rubric for the Literature-related Argumentative Writing Task.

8	7	6	5	4	3	2	1	Evaluation (0)
A sophisticated organizational framework is used to present the main arguments clearly and how the elements of arguments are related to one another. Interpretation and generalization that offers a thematic framing of the story with extensive, warranted and specific support from the text as well as other sources; multiple perspectives are considered; use of elaborated world knowledge and text evidence; may also include counter-claims that anticipate other interpretations.	A sophisticated organizational framework is used to present the main arguments clearly and how the elements of arguments are related to one another. Interpretation with extensive support that is warranted by and explained with elaborated detail from the text; may also include counter-claims that anticipate other interpretations.	An organizational framework is used to present the main arguments clearly and how the elements of arguments are related to one another. Interpretation with extensive support or evidence in the form of quotations or retelling of the story to support a claim.	A weak organizational framework makes it difficult to follow the arguments and how the elements of arguments are related to one another. Interpretation with some support or evidence in the form of quotations or retelling of the story to support a claim.	Lack of an identifiable organizational framework; A series of claims or warrants exists with minimal interpretations.	Retelling with elaborated details from the text with an interpretive tag.	Retelling with more elaborated details from the text.	Retelling with little elaboration.	Writer judges the quality of the work, character’s behavior, idea or author’s vision of the world; content may not relate to the prompt.

Depending on the extent to which students’ retelling was elaborated by interpretive tags, the retelling level was further classified into three sub-levels, labeled as Level 1–3 in the rubric. Interpretation refers to the writer going beyond what is in the text to justify motivations for characters or the meaning of the unfolding plot and to interpret the writer/reader motives. A high quality literary interpretation contains (a) a sophisticated organizational framework, defined as an argument structure that allowed the reader to follow the presentation of claims, evidence, warrant, and reasons, as well as the writer’s central claim and conclusions; (b) thematic framing, defined as a framework developed by the writer to make conceptual distinctions and to develop ideas that are key to what the writer wants to communicate, such as the theme of lost innocence, coping with loss, etc.; (c) multiple perspectives, referring to the writerly moves that rely on more than one point of view in understanding a character or theme or issue. Depending on how well the writer argued for his or her thematic framework and coordinated among multiple perspectives, the interpretation level was further classified into two sublevels (Level 7 and 8) based on the extent to which the three criteria were met. Essays that moved beyond retelling but did not satisfy the three criteria of interpretation were classified into Level 4, 5, or 6. These three intermediate sub-levels demonstrated the writer’s potential to shift their literature-related argumentative writing from retelling to interpretation. Essays were evaluated independently by two professional raters blind to study condition. The two raters and the research team met twice to go through 10 randomly selected pre- and post-essays. Based on the discussions, the team modified the scoring rubrics and assigned another batch of randomly selected essays (*n* = 38, 15%) from the current data set to the two raters for inter-rater reliability check. Adequate inter-rater reliability (Krippendorff’s α = 0.77) was obtained, and disagreements were resolved through discussions.

### Data analysis approaches

As shown in Table 3, the proportions of missing values in the key variables of the current study (i.e., argumentative writing performance, composites of self-efficacy for writing, composites of writing beliefs) ranged from 8.6 to 30.9%. The proportions of missing values in students’ demographic variables ranged from 0 to 8.3%. Little’s Missing Completely at Random test suggested that data were missing completely at random ( $\chi_{\left(78\right)}^{2}$ =80.727, *p* = 0.394). Even though the probability of missingness on a variable was unrelated to other measured variables and was unrelated to the variable with missing values itself, the maximum proportion of missing data for any variable was high (30.9%). To avoid producing biased results and to capitalize on all of the available information, we handled missing data using the Full Information Maximum Likelihood approach (FIML, Enders, 2001) in Mplus 8 (Muthén and Muthén, 1998/2017). The proportion of variance explained at the classroom level (i.e., intraclass coefficients) for argumentative writing and motivational beliefs ranged from 0 to 41%. However, the number of classrooms (i.e., level 2 clusters, *n* = 14) was not sufficient for Mplus to provide trustworthy estimates and satisfactory model fit in a TYPE = TWOLEVEL model. To account for interdependency of the nested data, we used TYPE = COMPLEX under the ANALYSIS command in conjunction with CLUSTER = Classroom in the VARIABLE command in Mplus. These commends will correct standard error biases using a sandwich estimation procedure (Berger et al., 2017). In addition, we reported standardized *β* to provide an estimate of effect size. To address the first research question (how students in the DLA and CR classrooms differ in their argumentative writing and motivational beliefs), we conducted multiple regressions and corrected for biased standard errors using TYPE = COMPLEX and CLUSTER = CLASSROOM. Post-test outcomes were predicted by instructional approach (DLA vs. CR), controlling for pre-test outcomes, gender, grade level, and academic track. To address the second research question (how the relationships between motivational beliefs and argumentative writing differ between the DLA and CR groups at the post-test), we conducted multigroup structural equation modeling (SEM) to examine whether the model structure was the same across groups. In the multigroup SEM process, we first tested an unconstrained multigroup SEM to explore potential path coefficients that might be different between the DLA and CR groups. We then compared it with a constrained model in which all path coefficients were set to be equal between the two groups to test model invariance. Finally, based on the constrained model, we release one set of path coefficients and assessed changes in the model fit between the unconstrained model and the constrained model. For the multigroup SEM, we did not test measurement invariance because (a) measurement invariance had been confirmed at the item level (see the Measure section), and (b) a latent construct with only two items is likely to be under-identified with negative degree of freedom during the measurement invariance test. The indirect effect of writing self-efficacy was tested using the Model Indirect command. A parametric bootstrapping approach was used to estimate standard errors of all the parameters and the indirect effects from writer beliefs to argumentative writing through writing self-efficacy (with 1,000 draws; MacKinnon et al., 2002; Shrout and Bolger, 2002).

**Table 3:** Means and standard deviations of writing motivation and argumentative writing performance.

Variable	Pre-test		Post-test	
	*n*	*M* (SD)	*n*	*M* (SD)
**Dialogic literary argumentation (*n* = 155)**				
Transactional writer beliefs	145	3.79 (0.54)	126	3.83 (0.63)
Transmissional writer beliefs	145	2.59 (0.67)	126	2.66 (0.75)
Self-efficacy for writing	145	3.63 (0.68)	126	3.59 (0.75)
Self-efficacy for argumentative writing	145	3.82 (0.67)	126	3.71 (0.69)
Argumentative writing performance	137	4.80 (1.73)	108	5.98 (1.63)
**Close reading (*n* = 123)**				
Transactional writer beliefs	109	3.76 (0.59)	97	3.80 (0.64)
Transmissional writer beliefs	109	2.69 (0.62)	97	2.75 (0.795)
Self-efficacy for writing	109	3.24 (0.82)	97	3.51 (0.69)
Self-efficacy for argumentative writing	109	3.47 (0.75)	96	3.65 (0.58)
Argumentative writing performance	107	4.77 (1.28)	84	5.29 (1.16)

## Results

### Exploratory analysis on pre-post differences, baseline equivalence, and correlations

The means and standard deviations of self-efficacy for writing, self-efficacy for argumentative writing, transactional writer beliefs, transmissional writer belief, and literature-related argumentative writing performances (hereafter called argumentative writing) are presented in Table 3. Paired t-tests of the pre-post changes in argumentative writing and motivational beliefs showed that, for the DLA group, students showed a greater argumentative writing performance (*t* = 10.00, df = 96, *p* < 0.001), a decrease in self-efficacy for argumentative writing (*t* = −1.99, df = 118, *p* < 0.05), and no changes in writer beliefs or self-efficacy for writing from pre-test to post-test. The CR group also showed greater argumentative writing (*t* = 2.58, df = 75, *p* < 0.01) and self-efficacy for writing (*t* = 2.6, df = 87, *p* = 0.01). There was no change in self-efficacy for argumentative writing or writer beliefs from pre-test to post-test in the CR group.

Table 4 presents multiple regressions of pre-test variables using Type = Complex and Cluster = Classroom, controlling for gender, grade, and academic track (1 = AP, 0 = CP). The results showed that the DLA and CR groups did not differ in argumentative writing performance and writer beliefs at the pre-test. However, the DLA group showed slightly higher self-efficacy for writing and self-efficacy for argumentative writing than the CR group. Therefore, the baseline equivalence assumption was met except for writing self-efficacy.

**Table 4:** Standardized coefficients and bootstrapped standard errors from multiple regression models of pre-test writing self-efficacy, writer beliefs, and argumentative writing performance (with FIML estimation).

	T1 self-efficacy for writing	T1 self-efficacy for argumentative writing	T1 transactional writer beliefs	T1 transmissional writer beliefs	T1 argumentative writing
	*β (SE)*	*β (SE)*	*β (SE)*	*β (SE)*	*β (SE)*
Gender (1 = Female)	−0.06 (0.06)	−0.11 (0.06)	−0.27^***^ (0.06)	0.15^**^ (0.06)	−0.14^**^ (0.04)
Grade Level	−0.05 (0.07)	−0.05 (0.05)	−0.05 (0.05)	−0.02 (0.04)	0.17 (0.11)
Academic Track (1 = AP, 0 = CP)	0.20^***^ (0.05)	0.21^***^ (0.07)	0.26 (0.07)	−0.39^***^ (0.05)	0.48^***^ (0.14)
Instruction (1 = DLA, 0 = CR)	0.19^*^ (0.08)	0.17^*^ (0.08)	−0.08 (0.07)	0.03 (0.06)	−0.07 (0.12)

Model fit indices are not reported because these models are saturated.

The correlations among these variables are presented in Table 5. Self-efficacy for writing and self-efficacy for argumentative writing were highly correlated (pre-test: *r* = 0.78, *p* < 0.001; post-test: *r* = 0.77, *p* < 0.001). Based on this result, a latent construct of writing self-efficacy will be estimated in the later structural equation models based on the two subscales of writing self-efficacy. Self-efficacy for writing and argumentative writing were positively correlated with transactional writer beliefs and were negatively or not associated with transmissional writer beliefs at the pre- and post-tests. Self-efficacy for writing and argumentative writing were positively associated with students’ argumentative writing at both time points, although the correlation between self-efficacy for writing and argumentative writing at the post-test was not significant. Transactional and transmissional writer beliefs were negatively correlated at the pre-test and did not correlate with each other at the post-test.

**Table 5:** Pearson’s correlations between writing motivation and argumentative writing performance at pre- and post-tests.

		Pre-test					Post-test				
		1	2	3	4	5	6	7	8	9	10
Pre-test	1. Self-efficacy for writing										
	2. Self-efficacy for argumentative writing	0.78^***^									
	3. Transactional writer beliefs	0.33^***^	0.33^***^								
	4. Transmissional writer beliefs	−0.26^***^	−0.22^***^	−0.11							
	5. Argumentative writing performance	0.25^***^	0.26^***^	0.20^**^	−0.36^***^						
Post-test	6. Self-efficacy for writing	0.44^***^	0.32^***^	0.24^***^	−0.15^*^	0.14^*^					
	7. Self-efficacy for argumentative writing	0.36^***^	0.39^***^	0.24^***^	−0.19^**^	0.11	0.77^***^				
	8. Transactional writer beliefs	0.24^**^	0.18^**^	0.50^***^	−0.10	0.27^***^	0.40^***^	0.44^***^			
	9. Transmissional writer beliefs	−0.27^***^	−0.30^***^	−0.23^***^	0.44^***^	−0.32^***^	0.06	−0.001	0.01		
	10. Argumentative writing performance	0.37^***^	0.35^***^	0.11	−0.37^***^	0.64^***^	0.15	0.20^**^	0.23^**^	−0.27^***^	

*p < 0.05, **p < 0.01, ***p < 0.001.

Transactional writer beliefs were positively associated with argumentative writing at both time points, but transactional writer beliefs at the pre-test was not associated with argumentative writing performance at the post-test. Transmissional writer beliefs and argumentative writing performance were negatively correlated at the pre- and post-tests; transmissional writer beliefs at the pre-test was also negatively associated with argumentative writing at the post-test.

### DLA versus CR in argumentative writing and motivational beliefs

To address the first research question, we fit the data with multiple regressions in Mplus. In separate models, writing motivation and argumentative writing at the post-test were predicted by instructional approach (DLA vs. CR), controlling for pre-test scores, gender, grade level (9th, 10th, 11th, 12th), and academic track (1 = AP, 0 = CP). A latent variable called writing self-efficacy was estimated by self-efficacy for writing and self-efficacy for argumentative writing at the pre- and post-tests. When predicting transactional or transmissional writer beliefs at the post-test, both types of writer beliefs at the pre-test were included in the models to control baseline differences.

As shown in Table 6, instruction (DLA vs. CR) significantly predicted post-test argumentative writing (*β* = 0.24, SE = 0.11, *p* < 0.05), controlling for pre-test argumentative writing and covariates. This suggests that the DLA group demonstrated more growth in argumentative writing than the CR group. The instruction effect was not significant in the models of writing self-efficacy, transactional writer beliefs, or transmissional writer beliefs, suggesting that students receiving DLA or CR did not differ in the level of change in writing self-efficacy or writer beliefs from pre- to post-tests. Students at a higher grade level tended to show higher writing self-efficacy than students at a lower grade level in high school (*β* = 0.24, SE = 0.07, *p* < 0.001). Interestingly, students in the AP English class had lower writing self-efficacy (*β* = −0.21, SE = 0.08, *p* < 0.01) and lower transmissional writer beliefs (*β* = −0.12, SE = 0.06, *p* < 0.05) than students in the CP English class. Gender did not predict any outcome variable at the post-test.

**Table 6:** Standardized coefficients and bootstrapped standard errors from multiple regression models of post-test writing self-efficacy, writer beliefs, and argumentative writing performance (with FIML estimation).

	T2 writing self-efficacy	T2 transactional writer beliefs	T2 transmissional writer beliefs	T2 argumentative writing
	*β (SE)*	*β (SE)*	*β (SE)*	*β (SE)*
Gender (1 = female)	−0.07 (0.06)	−0.07 (0.07)	0.10 (0.07)	−0.06 (0.05)
Grade level	0.24^***^ (0.07)	0.13 (0.09)	0.06 (0.07)	0.16 (0.12)
Academic track (1 = AP, 0 = CP)	−0.21^**^ (0.08)	−0.03 (0.06)	−0.12^*^ (0.06)	0.09 (0.13)
Instruction (1 = DLA, 0 = CR)	0.03 (0.05)	0.06 (0.08)	0.02 (0.08)	0.24^*^ (0.11)
T1 Writing self-efficacy	0.54^***^ (0.08)			0.20^**^ (0.07)
T1 Transactional writer beliefs		0.48^***^ (0.07)	−0.15^**^ (0.06)	−0.08 (0.09)
T1 Transmissional writer beliefs		−0.03 (0.08)	0.37^***^ (0.05)	−0.09 (0.06)
T1 Argumentative writing				0.45^***^ (0.10)
$\chi_{\left(df\right)}^{2}$	20.162 (13)	5.78 (5)	5.78 (5)	3.85 (7)
RMSEA	0.05	0.02	0.02	0.00
CFI	0.99	0.98	0.99	0.99
SRMR	0.08	0.06	0.06	0.01

RMSEA = root mean square error of approximation; CFI = comparative fit index; SRMR = standardized root mean square residual; T1 = pre-test, T2 = post-test.

### DLA versus CR in the relationships between argumentative writing and motivational beliefs

**Figure 1:**
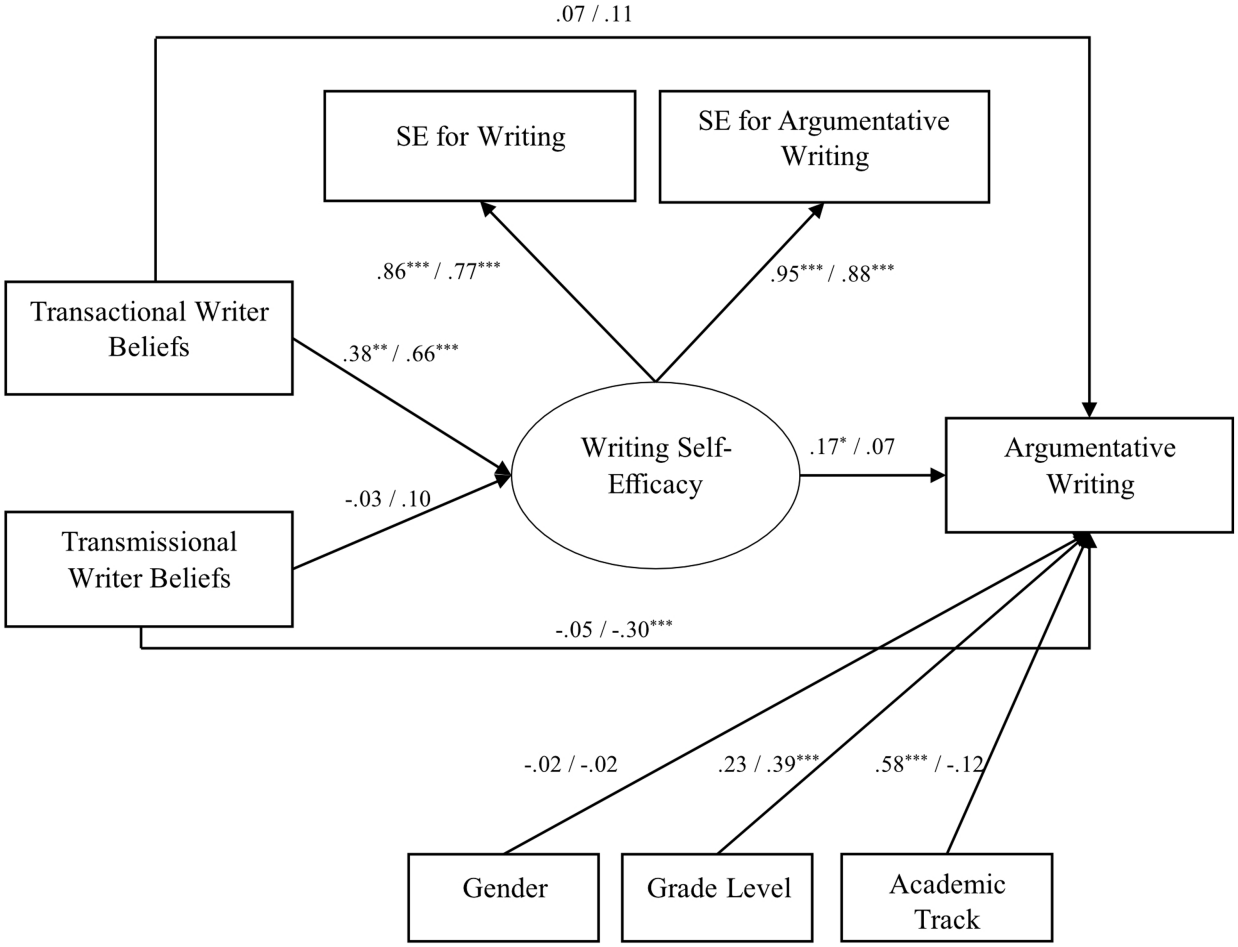
Unconstrained multigroup structural equation model (SEM) representing the relationship between writer beliefs, writing self-efficacy, and argumentative writing performance at the post-test. Standardized coefficient are reported. Each path is associated with two coefficient. The coefficient on the left-hand side were from the DLA model, and the coefficient on the right-hand side were from the CR model (DLA/CR). SE, self efficacy. ^*^p < 0.05, ^**^p < 0.01, ^***^p < 0.001.

Figure 1 presents a conceptual model in which argumentative writing is associated with writing self-efficacy and the two writer beliefs at the post-test, and writing self-efficacy mediates between writer beliefs and argumentative writing. To address the second research question, we tested whether this model structure differed between the DLA and CR groups. Control covariates included gender, grade level, and academic track.

An unconstrained multigroup SEM in which all path coefficients were allowed to be freely estimated had a good model fit ( $\chi_{\left(df=23\right)}^{2}$ =27.81, *p* = n.s., RMSEA = 0.04, CFI = 0.99). Comparatively, a constrained multigroup SEM in which all path coefficients were set to be equal between the two groups showed an acceptable model fit ( $\chi_{\left(df=31\right)}^{2}$ = 63.461 *p* = n.s., RMSEA = 0.09, CFI = 0.92). Three indicators were considered in making model comparisons: a likelihood ratio test comparing Chi-square differences between two models, and the level of changes in RMSEA and CFI. If a likelihood ratio test is significant, RMSEA increases by 0.015 or greater, and CFI decreases by 0.01 or greater from unconstrained to constrained models, these indicators suggest that the constrained model has poorer model fit (Chen, 2007). The three indicators ( $\Delta \chi_{\left(\Delta df=8\right)}^{2}$ = 35.66, *p* < 0.001, ΔRMSEA = 0.05, ΔCFI = 0.07) suggests that the unconstrained model had a better model fit than the constrained model. One or more path coefficients might vary between the DLA and CR groups.

To identify which path(s) were not equivalent between groups, we closely examined the unconstrained multigroup SEM presented in Figure 1. For the DLA group, the association between transactional writer beliefs and writing self-efficacy at the post-test was significant (*β* = 0.38, SE = 0.12, *p* < 0.01), but the association between transmissional writer beliefs and writing self-efficacy was not significant. Writing self-efficacy was positively correlated with argumentative writing (*β* = 0.17, SE = 0.07, *p* < 0.05). The two types of writer beliefs did not correlate with argumentative writing. For the CR group, the association between transactional writer beliefs and writing self-efficacy at the post-test was significant (*β* = 0.66, SE = 0.07, *p* < 0.001), and the association between transmissional writer beliefs and writing self-efficacy was not significant. Writing self-efficacy and transactional writer beliefs did not predict argumentative writing, but transmissional writer beliefs were negatively associated with argumentative writing (*β* = −0.30, SE = 0.10, *p* < 0.001). The indirect effects from transactional or transmissional writer beliefs to argumentative writing through writing self-efficacy were not significant for the DLA or the CR group.

The unconstrained model suggested that the DLA and CR groups might differ in the association between writing self-efficacy and argumentative writing, and the association between transmissional writer beliefs and argumentative writing. We subsequently conducted an invariance test such that only the direct path from writing self-efficacy to argumentative writing was allowed to vary between the groups. A likelihood ratio test comparing this relatively unconstrained model (M2 in Table 7) and the constrained model (M1) was not significant. We conducted another invariance test such that only the direct paths from transactional and transmissional writer beliefs to argumentative writing were allowed to be different between the two groups. A likelihood ratio test comparing this unconstrained model (M3 in Table 7) to the most constrained model (M1) was marginally significant ( $\Delta \chi_{\left(\Delta df=2\right)}^{2}$ = 5.68, *p* = 0.058), ΔRMSEA = 0.01, ΔCFI = 0.01. These indicators suggest that the pathways between writer beliefs and argumentative writing might be marginally different between DLA and CR groups. Specifically, transmissional writer beliefs seem to be more negatively associated with argumentative writing for the CR group than for the DLA group, while the association between transactional writer beliefs and argumentative writing might be more identical between the groups (Figure 1). In these models, the indirect effects from transactional or transmissional writer beliefs to argumentative writing through writing self-efficacy were not significant for the DLA or the CR group.

**Table 7:** Model comparisons between different multigroup structural equation models.

Model	Freed path coefficients	$\Delta \chi_{\left(\Delta df\right)}^{2}$	RMSEA	CFI	Model comparison	$\Delta \chi_{\left(\Delta df\right)}^{2}$	ΔRMSEA	ΔCFI
M1	None	63.46 (31)	0.09	0.92	M1	35.65 (8)^***^	0.05	0.07
M2	Writing self-efficacy ➔ Argumentative writing	61.85 (30)	0.09	0.92	M2	1.61 (1)	0.00	0.00
M3	Transactional writer beliefs ➔ Argumentative writing Transmissional writer beliefs ➔ Argumentative writing	57.78 (29)	0.08	0.93	M2	5.68 (2)^*^	0.01	0.01

M1 = constrained model, M2 and M3 were the same as M1 except for the freed path coefficients listed in the table. ^*^p < 0.05, ^**^p < 0.01, ^***^p < 0.001.

## Discussion

Compared to Close Reading, a method that varies in practice and that has shown promise in promoting students’ literacy repertoires as well as metadiscoursal awareness (Fisher and Frey, 2012; Beers and Probst, 2013; Snow and O’connor, 2016; Catterson and Pearson, 2017), our findings suggest that Dialogic Literary Argumentation can add value to Close Reading to improve high school students’ literature-related argumentative writing. This may especially be the case when DLA meaningfully includes the reader’s background, worldview, activities, and sociocultural context in the sensemaking process. These are key elements for models of writing that emphasize the communal aspects of writing (Graham, 2018), contrasting with CR’s primary focus on the “four corners of the text” (Coleman and Pimentel, 2012). Although the DLA and CR groups did not differ in motivational beliefs, post-test motivational beliefs and argumentative writing performance seem to be more positively correlated in the DLA group than the CR group. Based on the best-fitting unconstrained multigroup SEM model (Figure 1), transactional writer beliefs were positively correlated with writing self-efficacy in both groups, but writing self-efficacy was more positively correlated with argumentative writing and transmissional writer beliefs less negatively correlated with argumentative writing in the DLA group than the CR group.

Although the mechanisms of change underlying DLA are yet to be systematically explored, one plausible explanation of the added value of DLA in fostering students’ literature-related argumentative writing may be its deliberate emphasis on social, political, and historical context and on writing within community (Graham, 2018). On top of reading and re-reading literary texts to construct meaning out of the text through the transaction between the reader and the text, DLA focuses on constructing and reconstructing literary understanding through multiple perspectives, arguing to understand literary themes within the context of students’ own lives as well as the broader social world, and interactionally construct social relations among and between themselves to form a more informative dialectic through alternative arguing. These instructional principles might have made DLA more effective in connecting students with the literature in personally and culturally meaningful ways, triggering the right form of motivational beliefs about writing, and facilitating them to shift from retelling to argumentative interpretations of literature in students’ writing.

Even though paired t-tests suggest that the DLA group showed a slight decrease in self-efficacy for argumentative writing and the CR group showed a slight increase in self-efficacy for writing, the degree of change did not differ between the two groups. Both instructional approaches significantly enhanced students’ argumentative writing performance, but contrary to our predictions, neither condition significantly changed students’ motivational beliefs. Other studies have found similar results where student performance improves, but motivation remains unchanged (Graham et al., 2005; Harris et al., 2006). An exception to this is the Concept-Oriented Reading Instruction (CORI) program (Guthrie et al., 2004). This program was likely successful due to its five approaches to enhancing student motivation: knowledge content goals, student choice, hands on experiences, interesting texts, and student collaboration.

A possible explanation for our findings is that there could have been a lagging effect on motivational beliefs that were not measured in the current study. That is, motivational beliefs might not change until students have the opportunity to see and reflect on their own improved outcomes (Halper et al., 2018). Existing findings also indicate that changes in students’ motivational profiles (i.e., patterns of motivation) during school years tend to show variabilities across subgroups of individuals (Hayenga and Corpus, 2010; Gillet et al., 2017; Xie et al., 2022). A person-centered approach might be used in future research to identify students’ motivational profiles in argumentative writing and how students shift among these profiles over time as a function of instructional practices.

Transactional writer beliefs were predictive of writing self-efficacy in both DLA and CR conditions. This result supports the design principles of DLA and CR that meaning is actively constructed by learners in the process of reading and writing. However, the indirect effect of transactional writer beliefs on argumentative writing performance through writing self-efficacy was not significant in the multigroup SEM. Our original hypothesis was that when students hold the beliefs that meaning is not external to the writer but rather is actively constructed by the writer, such beliefs may motivate students to actively learn to write during English language arts instruction. The active learning process may help students evaluate their own competence about writing or argumentative writing more positively, which then fosters their literature-related argumentative writing performance. Unfortunately, our hypothesis was not fully supported by the current study. One possible explanation is that the argumentative essay is a challenging genre to write and may not be predicted by students’ writing self-efficacy unless the level of self-efficacy is high. DLA may have a greater promise than CR (as a text-centered only practice) to establish a transactionally dialogic environment to nurture competent writers in the classroom, although the current evidence is not robust enough to support this claim. In addition, high school students are more used to operating out of the transmissional beliefs framework in schools, considering schools’ emphases on preparing for and performing well on state mandated standardized tests, a pattern for which Applebee and Langer (2013) have raised significant concerns. The DLA approach may have been one of the few instructional practices in schools that encourage students to operate out of a transactional belief system. Students in the DLA group thus might have had to constantly reconcile these conflicting beliefs, which might have dampened the links between writer beliefs and writing self-efficacy or argumentative writing performance.

Interestingly, transmissional writer beliefs showed a negative correlation with argumentative writing performance in the CR group and a null relationship with argumentative writing performance in the DLA group in the unconstrained model. The pathways between writer beliefs and argumentative writing were marginally significant between the two groups. We conjecture that DLA may have functioned as a buffer against the negative influence that transmissional beliefs had on students’ argumentative writing performance. High-quality literature-related argumentative writing requires that writers develop a sophisticated organizational framework of writing that presents a thematic framing of the literature, warranted support of arguments from multiple sources, and elaborated world knowledge. Such expectations contradict the transmissional writer beliefs that meanings are given and transmitted from the external world. While both the DLA and CR approaches advocate for learners’ active construction of meanings, DLA is specifically focused on argumentation as a social process by which students build on each other’s ideas toward more meaningful understanding of the text, its associated human conditions and worldview (Bloome et al., 2020). When students have the opportunity to engage in alternative arguing about literature with the teacher and peers (Newell et al., 2015), they gain greater sense of agency and flexibility to explore different ways of understanding and using literary texts to engage in their social worlds. This dialogic social process of DLA might lend support to students holding greater transmissional writer beliefs as they joined the arguing-to-learn endeavor with others, which then improved their argumentative writing.

Writing self-efficacy at the pre-test, which incorporated highly correlated genre-general and genre-specific self-efficacy for writing, was associated with literature-related argumentative writing at both pre- and post-tests. However, the multigroup SEM suggests that writing self-efficacy at the post-test was mildly associated with post-test argumentative writing in the DLA group and not associated with post-test argumentative writing in the CR group, although the group difference did not reach a statistical significance. The weakened association between writing self-efficacy and argumentative writing at the post-test explains the null mediation effect of writing self-efficacy on the relationships between writer beliefs and argumentative writing performance. A possible reason for the trending difference in the association between writing self-efficacy and argumentative writing performance between the DLA group and the CR group might be that the instructional practices of DLA centering on literary argumentation were more aligned with the design and expectations of the literature-related argumentative writing task, therefore maintaining the positive association between students’ self-perception of their genre-specific competence and their actual literature-related argumentative writing performance. However, both groups of students showed weakened associations between writing self-efficacy and argumentative writing at the post-test. It is likely that for students with lower self-efficacy for writing or argumentative writing, the level of instructional support that they received from DLA or CR had strengthened their competence for argumentative writing, which then weakened the link between writing self-efficacy and argumentative writing performance.

### Study limitations and future research directions

Despite the important findings, the current study is subject to several limitations. One limitation is that the participating teachers were recommended by building principals or other authority figures, and the criteria by which the principals made the recommendations were not completely clear. We were told that the teachers were recommended based on their reputations for teaching literature or writing. However, we cannot exclude the possibility of self-selection or other teacher characteristics such as work ethics. In addition, the study was limited to 14 teachers, and five of their classrooms were taught at the AP level. Even though we controlled the effect of academic track in all statistical models, students who took AP courses might not be representative of the general student populations due to the prerequisites and increases in rigor common to AP courses. The small group of teachers also limited our ability to explore potential teacher influences. For example, the majority of AP teachers have a master’s degree in the discipline they teach (Milewski and Gillie, 2002), which contrasts with the statistics that most high school teachers do not have a master’s degree in the discipline they teach (National Center for Educational Statistics, 2018).

Another limitation of this quasi-experimental study is the small number of classrooms and that classrooms were not randomly assigned to study conditions, even though baseline equivalence was met. The limited sample size prohibited us from testing more complicated models. Future research should consider a randomized control trial study that aims at recruiting teachers and students who are representative of the populations of the study region. Random assignment of classrooms to study condition would allow researchers to compare the relative strengths, weaknesses, and general applicability of DLA and CR more systematically. Furthermore, multilevel models should be conducted in future studies to corroborate the findings reported in this study.

The paired *t*-tests comparing students’ pre- and post-test performance, as well as the multiple regressions comparing instruction effects on writing motivations, did not support DLA as effective means to promote motivational beliefs about writing. Earlier studies (Pajares et al., 2000) suggested that writing motivation could potentially be an outcome of writing achievement, supporting Bandura’s (1997) theory that mastery experience is one major source of self-efficacy beliefs. Adding additional data points in a cross-lagged longitudinal study framework to examine the reciprocal relationships between motivational beliefs and argumentative writing performance would likely add important nuance to previous systemic reviews which found that there tended to be weak to moderate associations between writing attitudes and writing performance (Camacho et al., 2021).

Even though we considered genre specificity in writing self-efficacy, the extant literature of self-efficacy suggests that self-efficacy as a three-dimensional construct: conventions (i.e., transcribing ideas into writing), ideation (i.e., generating good ideas), and self-regulation (i.e., managing the cognitive, emotional, and behavioral aspects of writing; Rasteiro and Limpo, 2023). Future research can be done to further examine whether the links between writer beliefs, writing self-efficacy, and argumentative writing performance would vary by sub-dimensions of self-efficacy. In addition, the current study did not include other types of motivational beliefs informed by the Writer(s)-Within-Community Model (Graham, 2018) due to schools’ reluctance to give in additional instruction time. These motivational beliefs, such as value and utility of writing, interest in writing, reasons for engaging in writing, and beliefs about writing communities, can be further examined in the context of DLA and CR.

## Conclusion

The teaching and learning of writing in the secondary English language arts classrooms in the United States is at stake given evidence suggesting that students’ writing motivation decreases with age (Pajares et al., 2007) and writing performance (U.S. Department of Education, 2019) has shown a downward development trend. In addition, the mixed findings on the complex relationships between motivational beliefs and writing performance (Camacho et al., 2021) and a lack of effective approaches to promoting both writing motivation and performance in the classroom suggest a great need of research in these aspects. One major contribution of the current study is to document the complex relationships between writing motivation, argumentative writing, and instructional approaches in the context of high school English language arts classrooms. Overall, our study provides evidence to suggest that Dialogic Literary Argumentation, potentially when implemented with close reading, can strengthen students’ literature-based argumentative writing skills through a socially constructive and self-efficacious learning process. This work serves as the first step toward developing an intellectually and socially engaging dialogic writing instruction in secondary education.

## Data availability statement

The raw data supporting the conclusions of this article will be made available by the authors, without undue reservation.

## Ethics statement

The studies involving human participants were reviewed and approved by Behavioral and Social Sciences Institutional Review Board, The Ohio State University Office of Research. Written informed consent to participate in this study was provided by the participants’ legal guardian/next of kin.

## Author contributions

KF and T-JL made contributions to the conception and drafting of the manuscript, analyzing data, and revising it critically for important intellectual content. GN and T-JL contributed to seeking grant, developing the study design, and collecting data. GN contributed to critical revision of the manuscript. All authors contributed to the article and approved the submitted version.

## Funding

This research reported here was supported by a grant from the U.S. Department of Education, Institute of Education Sciences through grant 305A100786.

## Conflict of interest

The authors declare that the research was conducted in the absence of any commercial or financial relationships that could be construed as a potential conflict of interest.

## Publisher’s note

All claims expressed in this article are solely those of the authors and do not necessarily represent those of their affiliated organizations, or those of the publisher, the editors and the reviewers. Any product that may be evaluated in this article, or claim that may be made by its manufacturer, is not guaranteed or endorsed by the publisher.
